# Subwavelength hyperspectral THz studies of articular cartilage

**DOI:** 10.1038/s41598-018-25057-9

**Published:** 2018-05-02

**Authors:** Rayko I. Stantchev, Jessica C. Mansfield, Ryan S. Edginton, Peter Hobson, Francesca Palombo, Euan Hendry

**Affiliations:** 1 School of Physics, University of Exeter, Stocker Road, Exeter, EX4 4QL UK; 2 QinetiQ Limited, Cody Technology Park, Ively Road, Farnborough, GU14 0LX UK

## Abstract

Terahertz-spectroscopy probes dynamics and spectral response of collective vibrational modes in condensed phase, which can yield insight into composition and topology. However, due to the long wavelengths employed (λ = 300 μm at 1THz), diffraction limited imaging is typically restricted to spatial resolutions around a millimeter. Here, we demonstrate a new form of subwavelength hyperspectral, polarization-resolved THz imaging which employs an optical pattern projected onto a 6 μm-thin silicon wafer to achieve near-field modulation of a co-incident THz pulse. By placing near-field scatterers, one can measure the interaction of object with the evanescent THz fields. Further, by measuring the temporal evolution of the THz field a sample’s permittivity can be extracted with 65 μm spatial resolution due to the presence of evanescent fields. Here, we present the first application of this new approach to articular cartilage. We show that the THz permittivity in this material varies progressively from the superficial zone to the deep layer, and that this correlates with a change in orientation of the collagen fibrils that compose the extracellular matrix (ECM) of the tissue. Our approach enables direct interrogation of the sample’s biophysical properties, in this case concerning the structure and permittivity of collagen fibrils and their anisotropic organisation in connective tissue.

## Introduction

In the last two decades, THz radiation has attracted a lot of attention due its unique properties^1–3^. For example, there have been non-invasive inspections of semiconductor surfaces^4^, space shuttle panels^5^, electronics^6^, paintings^7^ and pharmaceutical tablets^8^. Unlike X-rays, the photon energies are non-ionizing, hence the great interest in using THz for biological tissue evaluation^9,10^ and also for cancer diagnosis^3,11^. Moreover, many low-frequency vibrational modes of biological molecules in aqueous media lie in this frequency range, allowing THz spectroscopy to identify and characterize inter-molecular bonding in amino acids^12^, sugars^13^, DNA^14^ and proteins^15^, as well as dynamics at biomolecule-water interfaces^16^ and in photoactive proteins^17^. There are also the THz investigations of corneal diseases by Taylor *et al*.^18^ and the diabetic foot studies by Hernandez-Cardoso *et al*.^19^. Furthermore, long-range collective vibrational modes, which mediate structural changes and the reaction coordinates critical to the function of active proteins^20^, normally manifest themselves at THz frequencies.

Whilst THz spectroscopy can readily identify such collective vibrational modes^21^ there are several difficulties, in addition the broadband nature of the resonances, in determining structural features of these systems. Firstly, samples have to be kept hydrated for normal biological function to be maintained, which is problematic due to the large THz absorption of water^22^. Secondly, owing to the long wavelengths employed (*λ* = 300 *μ*m at 1 THz), near-field approaches are generally required to get sub-mm resolution. However, invasive imaging techniques such as those involving scanning tips or apertures^23–25^ are not suited for biological applications. Furthermore, it is usually necessary to encapsulate biological samples to maintain hydration, severely restricting the resolution achievable by scanning tips or apertures, and the apertures themselves typically have a very strong frequency response^26^ making them unusable for spectroscopic applications. For these reasons, subwavelength spectroscopic THz measurements of biological samples^27–29^ have been plagued by problems, and biological imaging has, for the most part, been restricted to large structures such as organs^30,31^.

Apertureless near-field THz measurements offer an intriguing solution to many of these problems. In the approach previously described by refs^29,32^ by placing a sample directly onto a crystalline electro-optic crystal THz detector, the near-field THz radiation can be observed via a femtosecond optical detection pulse incident from the rear of the crystal. This strategy is highly advantageous for biological imaging as the crystal detector itself can be used to encapsulate the sample^29^, and an image is readily obtained by raster scanning the detection pulse. However, a major shortcoming of this approach^29,32^ is that the electro-optic crystal must be transparent for optical detection, hence the sample is exposed to the intense femtosecond visible pulse. Moreover, since the sample is in contact with the detector, this latter influences the reflection of the detection pulse, hence a measured image can be composed of both optical and THz responses, which may be of comparable magnitudes in biological samples.

**Figure 1:**
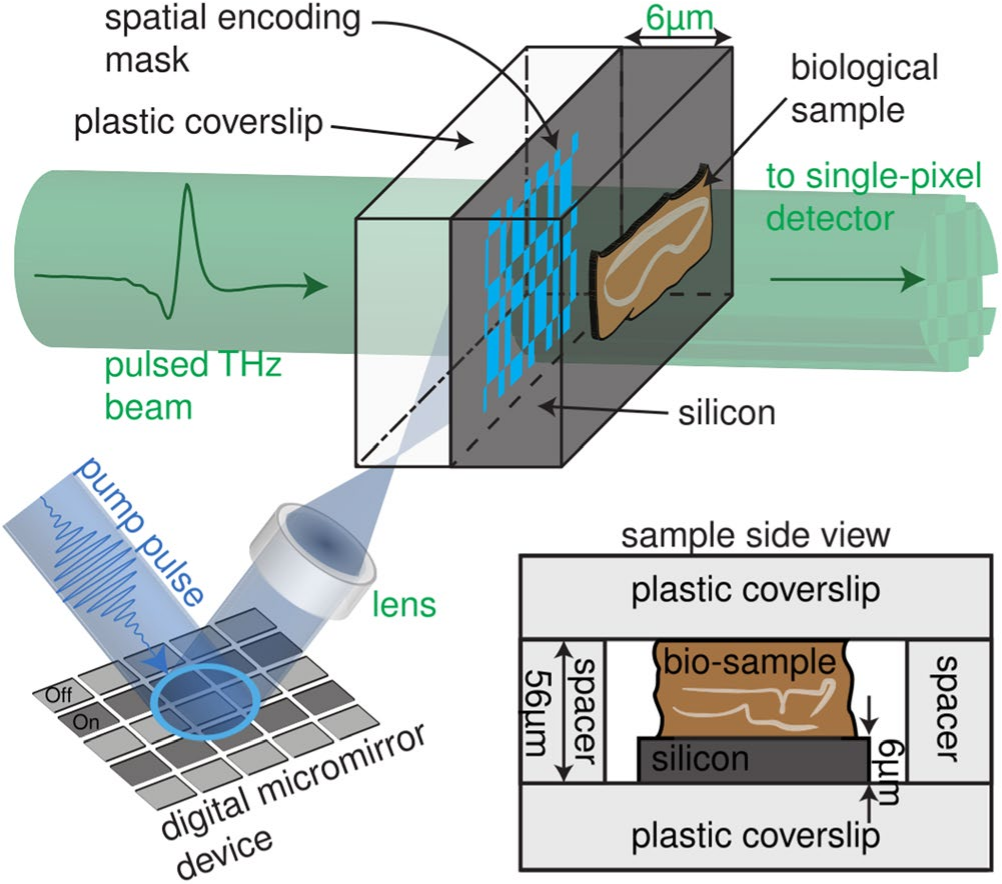
The experimental scheme: an optical pump pulse is spatially modulated and used to photoexcite a thin silicon wafer, which, in turn, transfers the spatial encoding mask onto a coincident THz pulse. The subsequent THz pulse is then passed through a biological sample onto a time-gated, single-element detector. By varying the arrival time of the electro-optic sampling pulse, we measure the full temporal trace of our THz waveform.

An alternative apertureless approach involves the use of a photoconductive modulator to spatially modify a THz beam^33^. Here, an optical pump beam is projected simultaneously with a THz beam onto a thin photoconductive modulator such as a semiconductor wafer (see below; Fig. 1), switching the THz material response from dielectric to conductor through electron-hole pair photoexcitation^34^. The photoconductive regions generated by the pump behave as scatterers for THz radiation in the vicinity of a sample, which is placed on the rear interface of the modulator. This approach offers several clear advantages: firstly, there is no mechanical raster scanning involved. Moreover, the spatial resolution of a sample placed directly after the modulator is determined primarily by the thickness of the photo-modulator^35^, and such sub-wavelength THz measurements have been achieved in a variety of solid state systems^35–37^. Furthermore, this approach enables Hadamard transform imaging, where binary intensity patterns spatially modulate a beam of radiation allowing the formulation of an image by analysis of the transmitted or reflected light^38,39^. Hadamard approaches can significantly improve image quality and acquisition times^35^, which proves particularly advantageous for imaging biological samples due to the rather problematic THz absorption of water therein.

Articular cartilage is a connective tissue composed of a dense extracellular matrix (ECM) rich in water, collagen and proteoglycans, with sparse specialised cells called chondrocytes^40^. It provides a smooth and lubricated surface for articulation and facilitates the transmission of loads through the distinctive regional orientation of the collagen fibrils, showing a change in alignment going from the articular surface through to deeper within the tissue. For this reason, cross-sections of articular cartilage are suitable candidates to test the capabilities of the THz imaging technique with polarization resolution. The thin superficial zone is made primarily of collagen fibrils aligned parallel to the articular surface, whilst the middle zone is composed of thicker collagen fibrils with an oblique alignment, and the deep zone consists of collagen fibrils aligned orthogonal to the articular surface^41^. Clinical conditions such as osteoarthritis and rheumatoid arthritis are characterized by degradation of the cartilage matrix, resulting in a disruption of the organised collagen structure^42^. Techniques that are able to detect changes in structure at the fibril level have potential for diagnosis of these pathologies.

In this article, we present a subwavelength THz measurement technique, based on the photoconductive modulator approach from refs^35–37^ which is applicable to histological sections of biological tissues. We project binary intensity patterns from a femtosecond laser source onto an ultrathin (6 μm-thick) photoconductive silicon wafer in order to modulate a coincident picosecond THz pulse. Cross-sections of healthy articular cartilage are placed on the rear interface of a silicon wafer for maximal near-field interaction. By varying the arrival time of the incident THz pulse and using time domain detection, we measure the full temporal evolution of the THz field. With both amplitude and phase of the scattered THz pulse determined, we are able to extract the frequency-dependent complex THz permittivity of our sample with subwavelength resolution. We show that the THz permittivity of articular cartilage, made essentially of type-II collagen, varies across tens to hundreds of micrometres depending on the protein fibril orientation. This demonstrates the advantage of our approach in mapping the micro-structure of anisotropic samples, previously unattainable using far-field approaches. Note that this technique, in transmission geometry, is only applicable to histologically sectioned samples and hence is only suitable for *ex-vivo* studies. However, we do point out that it may be possible to apply similar principles to study THz reflection from surfaces such as skin.

## Experimental Technique

Figure 1 illustrates the experimental setup (a more detailed schematic is presented in ref.^35^). We use a typical THz time domain spectrometer (THz-TDS) to launch and subsequently detect a THz pulse. Briefly: an amplified 800 nm (90 fs) Ti-Sapphire femtosecond laser running at a repetition rate of 1050 Hz, is used to power the THz-TDS using optical rectification and electro-optic sampling in ZnTe crystals for generation and detection of our terahertz pulses, respectively^43,44^. The femtosecond pulses also provide a third optical excitation beam with a fluence of $\sim 100\,\mu J/c{m}^{2}$. This pump pulse is spatially modulated via a digital micromirror device (DLP3000 with the DLP Lightcrafter from Texas Instruments) and a single lens so as to project an optical intensity pattern on the surface of a highly resistive silicon wafer (8000 Ω.*cm*, 6 *μm* thick). This projection is coordinated at the sample with the arrival of a THz beam. The biological sample, articular cartilage composed mainly of type II collagen fibrils, consists of 40 *μm*-thick histological cryosections of bovine cartilage (see Materials and Methods). The hydrated sample is placed on the rear interface of the photomodulator, which is in turn sandwiched between two optically transparent polystyrene coverslips to maintain sample hydration and structural integrity. The photoconductive properties of the silicon wafer allow one to optically render some regions opaque to THz radiation^34^, and scatter the incident THz light in the vicinity of the sample. Then, by measuring the far-field THz transmission for different spatial photo-excitation patterns, the near-field THz response of the object at different spatial locations can be obtained. Optimal signal-to-noise ratio is achieved via the use an orthogonal set of binary patterns derived from Hadamard matrices^38,39^ (see Materials and Methods). Moreover, by varying the relative arrival time of our electro-optic sampling pulse, we measure the full temporal evolution of the transmitted THz field with 100 fs temporal resolution. Combined with a reference scan taken in the absence of a sample, we are able to extract the frequency-dependent complex THz permittivity (see Materials and Methods for mathematical details) of the sample with a spatial resolution determined by the optical pattern on the photomodulator. We find that scatterers of size 65 *μm* to be sufficient to resolve the spatial variations of interest in the cartilage sample.

## Plane Wave Analysis

A standard approach to analyzing THz-TDS spectra is to extract the complex permittivity (or equivalent) via analysis of the Fresnel transmission equations^44^. However, this approach assumes a plane wave approximation, something that is questionable for the near field. In this section, we test the validity of such an approximation to our experimental approach.

**Figure 2:**
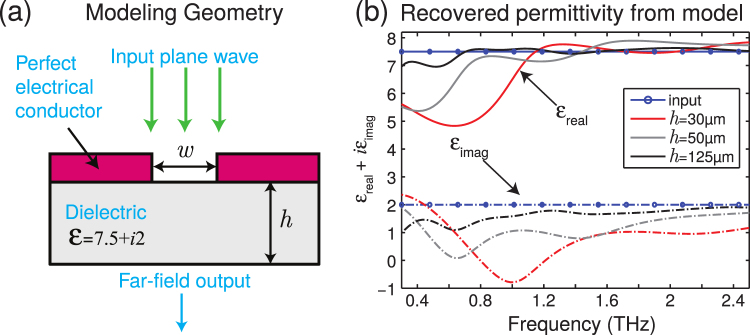
(**a**) Side view of the modelling geometry; a plane wave is incident upon a single aperture placed on top of a dielectric. (**b**) The permittivity recovered from our model for three different dielectric thicknesses of 30, 50 and 125 *μm*. The fields processed for the permittivity are due to the complex summation of the far fields transmitted through apertures of sizes from 40 to 700 *μm*.

We analytically model a system similar to that in our experiment (full mathematical details in supplementary information). In brief, we analyze the transmission through a single aperture in a conducting film in contact with a lossy dielectric layer of thickness *h*, as represented in Fig. 2a. Here, the region with the aperture is tailored to have similar transmissive properties to those of the experimental photomodulator, while the lossy dielectric is given a permittivity *ε*. We set the permittivity of the incident and transmitted regions to *ε*_*s*_ = 2.5, i.e. similar to that of the plastic coverslips encapsulating our sample. Using a modal matching model^37^ which assumes an incident THz plane wave, we simulate experiment by finding the transmitted far field for the two cases where *ε* = 7.5 + 2*i* (i.e. similar to our cartilage sample discussed below) and *ε* = 1 (representing our reference). To replicate the multi-aperture approach used in our experiment, we carry out a complex summation of fields transmitted through different sized apertures (see supplementary information). We then analyze the total transmitted fields via the approach outlined in the Materials and Methods section in order to extract the permittivity of the lossy dielectric layer. By comparing the extracted permittivity to that introduced in the model, we can assess the validity of the plane wave approximation.

In Fig. 2b, we plot the real and imaginary parts of the recovered permittivity versus frequency for three different sample thicknesses. We see that at higher frequencies, the recovered permittivity is generally very close to the input value used in the model. However, a greater discrepancy is found at lower THz frequencies, pronounced in both real and imaginary parts of the permittivity. This discrepancy arises from the presence of near fields, which are neglected in the plane wave approximation made to extract the permittivity. The longer decay lengths of the low frequency evanescent field components^45^ lead to a greater discrepancy than the high frequency fields. We also see that the thin samples exhibit greater discrepancy: for thinner samples, the amplitude of evanescent field components at the exit interface is larger. We discuss in more detail the origin of these effects in the supplementary information.

One should note that discrepancies due to the plane wave approximation are expected to be less severe in our experiment, owing to the much lower, finite conductivity of the photomodulator^35,37^, which will act to relax the aperture boundary conditions^46^ and reduce the amplitude of evanescent field components. Nevertheless, for sample thicknesses on the order of *μm* (such as those used in the experiment), one has to question the validity of the plane wave approximation at low THz frequencies. For this reason, we do not consider the very low frequency part of our spectra, below ~0.6 *TH*_*z*_. Note that for higher resolution images or thinner samples, one needs to develop a more elaborate analysis procedure, incorporating all near field effects, in order to reliably extract values of local permittivity.

## Results and Discussion

**Figure 3:**
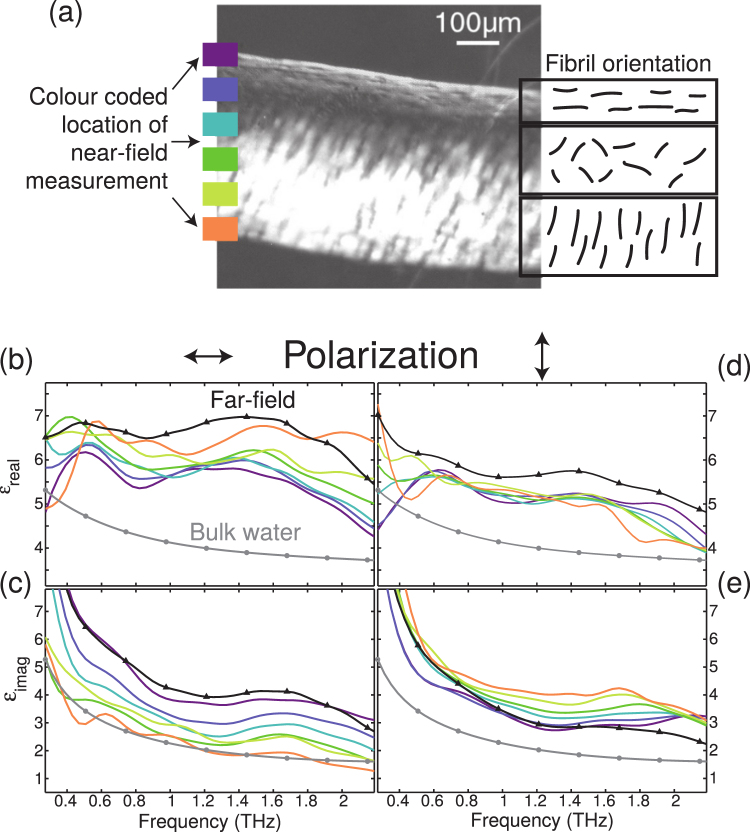
(**a**) Photomicrograph and schematic diagram of a cross-section of bovine articular cartilage taken with polarization microscope (Nikon Elclipse E200) at 45° to the articular surface. Boxes of different colour indicate locations from which THz measurements were taken. (**b**–**e**) Real and imaginary part, respectively, of the sample’s dielectric function for horizontally (vertically) polarized incident THz beam. Bulk water data from fit parameters of ref.^22^. Note the colour coding of the lines correlates with in part (**a**), indicating the location on the sample from where THz measurements were performed. The raw THz data as well as absorption maps at 1 THz can be seen in the supplementary information section S2.

Figure 3a shows a photomicrograph of a cross-section of articular cartilage taken with a polarized visible light microscope. The sample contains three main regions with distinct orientations of the collagen fibrils, similar to samples studied previously with other imaging techniques^47,48^. In the superficial zone, collagen fibrils are aligned parallel to the articular surface. In the middle zone, the fibrils have an oblique arrangement, then ending orthogonal to their starting alignment in the deep zone, which presents high intensity of the transmitted polarized light. While articular cartilage has a collagen ultrastructure with spatial dimensions ~100 *nm*^49^ which cannot be resolved here, we concern ourselves primarily with resolving orientation of the collagen fibrils which also occurs on a subwavelength scale for THz radiation.

Figure 3b–e show the subwavelength THz response of cartilage measured with polarization parallel and perpendicular to the articular surface. Measurements were performed at discrete locations, from the superficial through to the deep zone, encompassing the different orientations of the collagen fibrils indicated in Fig. 3a. As a comparison, we also plotted the permittivity of the sample measured in the far field (i.e. a spatial average measured through the entire sample) and the permittivity of pure water (taken from ref.^22^). Note that water alone accounts for nearly 80% of the wet weight of articular cartilage^40^, and that, due to the THz diffraction limit, the far-field spatially averaged measurement is carried out over a sample length of ~0.5 *mm*, a length scale over which both the protein concentration and fibril orientation can be expected to vary substantially, owing to the heterogeneity of the biological sample on a micro-scale. The water spectral response shows a decreasing permittivity with increasing frequency^22^. However, both the spatially averaged and subwavelength THz response at all points across the depth of the cartilage exhibit broad features that are not apparent in the spectrum of pure water. Here, the broad peak at $\sim 1.5THz(50\,c{m}^{-1})$ in the real part of the permittivity spectrum ($\sim 1.7THz$ in the imaginary part) is not due to bulk water and hence, is a feature associated with hydration water and the fibrils themselves (note that the smaller oscillatory peaks in the spectrum are artefacts of the finite Fourier transform used in the analysis, depending on the temporal length of the THz measurement). Finally, the observation that the far-field permittivity is not the average of the near-field permittivities exists. Finally, we note that the far-field measured permittivity is not characteristic of the spatial average of the near-field permittivities. We believe there are two origins to this effect. Firstly, in a spatially inhomogeneous sample, the coherent averaging of transmitted fields is not expected to be representative of the spatially average permittivities themselves. Moreover, the THz spot size is larger than the sample itself, which makes any far-field measurement unreliable.

When we compare the cartilage’s local permittivity, measured as a function of the distance from the superficial zone to the deep layer, to the spatially averaged measurement, we see a number of striking traits. Firstly, for horizontal THz polarization (Fig. 3b,c), the real part of the THz permittivity *increases* going from the superficial to the deep zone (top to bottom in Fig. 3a), whilst the imaginary part *decreases*. This indicates that the sample is most polarizable when the THz field is oriented along the fibril direction, i.e. in the superficial zone, and suggests that the collagen fibrils have a THz frequency dipole moment oriented along their principal axis. This assignment is corroborated by measurements with THz polarization rotated by 90 degrees (Fig. 3d,e): here the spatial dependence of the permittivity is essentially reversed and the sample is most polarizable at a deep location where the THz field is oriented along the fibril axis. It is important to note that the variation between the two sets of measurements in Fig. 2 most likely arises from the response of two slightly different areas of the sample, and is indeed representative of the variation when measuring day to day in the lab, a problem arises due to an inherent difficulty in positioning the sample on such small length scales. Hyperspectral measurements of a second sample are shown for comparison in supplementary section S3, which exhibits similar features to the results presented here. It is important to note that slight variations in sample thickness or hydration level will lead to slightly different values for the extracted for the real and imaginary parts of the permittivity. This is a well-known problem in phase resolved measurements, since the optical thickness of a sample will determine to a large degree the phase of the transmitted wave. Nevertheless, we again observe a clear resonance at 1.6 THz in regions where the polarisation is aligned to the collagen fibril axis. It has been shown that proteins have low-frequency vibrational modes in the far-IR region^50^, as well as coupled solute–“solvent modes from the solvated solute^51^. For a biological tissue such as cartilage, both fibrous type-II collagen and water in proximity to the protein (i.e. hydration water), may contribute to the total THz response. Markelz *et al*. have shown that collagen (lyophilised powder) has a rapidly increasing absorbance with increasing frequency in the range 0.3 to $\sim 1.25THz$^14^. Our data are in line with those findings, and we speculate that this broad absorption band is associated with the intermolecular structure of collagen. The strong dependence of the spectral response upon the THz field polarization may be associated with (water-mediated) collagen interstrand coupling^14^, which is stronger when the fibrils are aligned parallel to one another. It is possible that such interstrand coupling could play an important role in stabilizing the collagen structure^52^. Alternatively, the alignment of the water network in the direction of the fibrils gives rise to the polarisation effect observed, and further studies of the localized polarization-sensitive THz response observed here could provide greater insight.

## Conclusions

We have demonstrated for the first time subwavelength hyperspectral THz imaging of articular cartilage using the photoconductive properties of a silicon photomodulator. We study articular cartilage, composed of collagen which is the most abundant structural protein in the human body, and find that its THz dielectric function varies on a sub-THz wavelength scale depending on collagen fibril orientation, which could be due to the presence of a THz dipole moment along the primary axis of the fibril or the collagen is birefringent. We point out that such a detailed observation is impossible to deduce from far-field measurements, demonstrating the value of this subwavelength approach in regards to the diagnosis of pathologies that alter the collagen structure. It is interesting to note that, since the fundamental imaging resolution limit of our measurement is determined by the diffraction of the optical pump pulse, we believe that our approach, where sub-micron resolution may even be possible, holds promise as a future microscopy tool with potential for applications in the biomedical sciences, even on subcellular scales. However, while the presence of a THz resonance in oriented regions of articular cartilage is certainly a promising observation, we acknowledge that it is as yet unclear whether this additional information could potentially be useful for diagnosis. Moreover, to implement such a THz imaging technique in real-world applications, improvements to both the data acquisition rates as well as the current costs of THz measurement systems will be required.

## Methods

### Sample preparation

Bovine metacarpophalangeal joint cartilage was obtained from a local abattoir and washed in phosphate-buffered saline (PBS; pH 7.4) before cryosectioning. A cartilage segment was immersed in Bright cryo-m-bed compound and frozen before cryosections were cut. Cross-sections of cartilage were cut perpendicular to the articular surface and analyzed. The geometry of the section was recorded in polarized light microscope images, obtained using a 10X objective on a standard polarized light microscope and a CCD camera (QImaging Retiga 2000R).

### Orthogonal patterns

We observe THz transmission via a single-element detector in the far-field. Hence, as mentioned in the main text, our sub-wavelength resolution is achieved by modulating our THz beam with different encoding patterns in the near-field of our sample. To achieve optimal signal-to-noise ratio, we use an orthogonal set of binary patterns derived from Hadamard matrices^38,39^. We now consider the construction of an *N*-pixel image Ψ; our *i*^th^ measurement, *ϕ*_*i*_, is the dot product of the object transmission function and the *i*^th^ mask configuration, mathematically expressed as

1 $${\varphi }_{i}=\sum _{j\mathrm{=1}}^{N}{w}_{ij}{\psi }_{j},$$

where *w*_*ij*_ holds the spatial information of the *i*^th^ mask and *ψ*_*j*_ is the *j*^th^ pixel of the image. This can be represented by the matrix equation Φ = *W*Ψ, where the rows of matrix *W* are reformatted into the projected masks. For invertible matrices *W*, the image vector Ψ can be obtained through matrix inversion Ψ = *W*^−1^Φ, which then has to be reshaped into a 2D matrix of pixel values. Further, the matrix equation Φ = *W*Ψ represents the image being expanded in some basis given by *W*. For this study, we use Hadamard matrices as the basis expansion, i.e. *W* is a Hadamard matrix of order *N*. A Hadamard matrix *H*_*n*_ is defined as an *n* × *n* matrix of +1 s and −1s with the property that the scalar product between any two distinct rows is 0 (each row is orthogonal to every other one). Thus *H*_*n*_ satisfies:

2 $${H}_{n}{H}_{n}^{T}={H}_{n}^{T}{H}_{n}=n{I}_{n},$$

where ${H}_{n}^{T}$ is the transpose of *H*_*n*_. This allows for easy image reconstruction based on ${H}_{n}^{-1}={H}_{n}^{T}/n$. Moreover, a Hadamard basis minimizes the mean square error of each pixel in the image^38^. Here, masks are created via the photoexcitation of silicon, thereby rendering some pixels opaque and leaving the rest transmissive. This means that the physical masks are composed of 1 s and 0 s whereas Hadamard matrices are made of +1 s and −1s. This prevents us from doing a fully orthogonal measurement. However, as is outlined in^39^, we can still perform such a measurement with our system. For this, we carry out sequential measurements of a mask directly followed by its inverse and record the difference in THz transmission via a lock-in amplifier. The signal acquisition time for each mask and its inverse is 100 ms. Note that the THz transmission is recorded within a 5 ps window after photoexcitation to minimize electron diffusion in the silicon photomodulator(see supplementary of ref.^35^) and subsequent smearing and broadening of spatial features.

### Calculating The Permittivity

To obtain the permittivity of a sample using THz-TDS, one typically performs two measurements: one measuring the temporal waveform transmitted through a sample and the other to obtain a reference waveform without the sample. However, we cannot assume a homogenous beam. For this reason, our reference is recorded for each pixel, performing the same measurement on the same system without the sample in place. After Fourier transformation of the time axis, one can divide signal by reference to obtain the frequency dependent amplitude transmission coefficients. These are then equated to the transmission functions of the system, calculated using the transfer matrix method^44^:

3 $$t=\frac{2\sqrt{{\varepsilon }_{i}}}{{M}_{21}+\sqrt{{\varepsilon }_{i}}{M}_{11}+\sqrt{{\varepsilon }_{f}}({M}_{22}+\sqrt{{\varepsilon }_{i}}{M}_{12})},$$

where *ε*_*i*_ and *ε*_*f*_ are the permittivities of the initial and final media, respectively, enclosing the multilayer system and *M* is a 2 × 2 matrix associated with the propagation through the entire multilayer system. This matrix is given by the product of the individual layer matrices, *M* ≡ *M*_1_*M*_2_*M*_3_…*M*_*N*_, describing the propagation through each layer. The characteristic matrix of the *j*^th^ layer, *M*_*j*_, with thickness *l*_*j*_ and dielectric function *ε*_*j*_ is given by

4 $${M}_{j}=[\begin{array}{cc}\cos \,{\beta }_{j} & \frac{-i}{\sqrt{{\varepsilon }_{j}}}\,\sin \,{\beta }_{j}\\ -i\sqrt{{\varepsilon }_{j}}\,\sin \,{\beta }_{j} & \cos \,{\beta }_{j}\end{array}],$$

where ${\beta }_{j}=\omega {l}_{j}\sqrt{{\varepsilon }_{j}}/c$ is the phase delay associated with light propagation inside the *j*^th^ layer. By equating the experimental amplitude transmission coefficients with (3), we can then solve for the permittivity of the sample as a function of space and frequency.

## Electronic supplementary material

Supplementary information
