# Supplementary material for: Subwavelength hyperspectral THz studies of articular cartilage
Source: Sci Rep. 2018 May 2;8:6924. doi: 10.1038/s41598-018-25057-9 (PMC5932036; doi:10.1038/s41598-018-25057-9)
Supplement: Supplementary file 1 — Supplementary information [file 41598_2018_25057_MOESM1_ESM.pdf]

Supplementary information: Subwavelength hyperspectral THz studies of articular cartilage

Rayko I. Stantchev[1,*], Jess Mansfield[1], Ryan S. Edginton[1], Peter Hobson[1,2], Francesca Palombo[1], and Euan Hendry[1]

[1]School of Physics, University of Exeter, Stocker Road, Exeter EX4 4QL, UK,
[2]QinetiQ Limited, Cody Technology Park, Ively Road, Farnborough, GU14 0LX, UK
[*]ris202@exeter.ac.uk

ABSTRACT

Terahertz spectroscopy probes the dynamics and spectral response of collective vibrational modes in condensed phase, which can in turn yield insight into composition and topology. However, due to the long wavelengths employed (λ=300μm at 1THz), diffraction limited imaging is typically restricted to spatial resolutions just below a millimeter. In this work, we demonstrate a new form of subwavelength hyperspectral, polarization-resolved THz imaging which employs an optical pattern projected onto a 6μm-thin silicon wafer to achieve near-field modulation of a co-incident THz pulse. By placing a near-field scatterer, one can measure the interaction of object with the evanescent THz fields. Further, by measuring the temporal evolution of the THz field the THz permittivity of a sample can be extracted with 65μm spatial resolution due to the presence of evanescent fields. Here, we present the first application of this new approach to articular cartilage. We show that the THz permittivity in this material varies progressively from the superficial zone to the deep layer, and that this correlates with a change in orientation of the collagen fibrils that compose the dense extracellular matrix (ECM) of the tissue. Our approach enables direct interrogation of the biophysical properties of the sample, in this case concerning the structure and permittivity of collagen fibrils and their anisotropic organisation in connective tissue.

S1 Method of Modal Matching

Field definitions and boundary conditions

In this section we outline a full wave modal matching solution which describes the evolution of field components emanating from our photomodulator, and their subsequent effects during permittivity extraction. The time dependent components of the fields ($e^{i\omega t}$) have been omitted for clarity. We begin by selecting four regions within the cartilage sample along the z-axis direction. Region 1 extends to the half space on the incident side of silicon photomodulator and sample. Using the angular spectrum representation[1,2] we have a normally incident plane wave and a reflected component that is a superposition of plane waves propagating away from the sample, written

$$E_{1x} = e^{ik_{1,z}(0)z} + \int_{-\infty}^{\infty} A_r(v_x) e^{-ik_{1,z}(v_x)z} e^{iv_x x} \mathrm{d}v_x, \tag{1}$$

where v_x is the directional wavevector in x, $A_r(v_x)$ is a spectral amplitude function and $k_{1,z}(v_x) = \sqrt{(n_1 k_0)^2 - v_x^2}$. In the region describing our silicon modulator (region 2) our fields are represented by the modes of a cavity, defined by unexcited dielectric sections surrounded by photoexcited metallic regions. Note that the metallic regions of this cavity are assumed to be perfectly metallic (i.e. infinite conductivity) in order to simplify the boundary conditions. For simplicity, we choose polarization perpendicular to our cavity, thus the electric field parallel to the interfaces of the conducting sections will be zero. These boundary conditions will thus dictate that the fundamental mode of our cavity be described by a rectangle function. This is written

$$E_{2x} = \left(G_1 e^{ib_z z} - G_2 e^{-ib_z z} \right) \mathrm{rect}\left(\frac{x}{w} \right) \tag{2}$$

where $b_z = n_2 k_0$ is the wave vector inside the cavity, w is width of cavity. In region 3, containing the sample, we have two sets of wave superpositions, each travelling in opposite z directions, written

$$E_{3x} = \int_{-\infty}^{\infty} F_1(v_x) e^{ik_{3,z}(v_x)z} e^{iv_x x} \mathrm{d}v_x - \int_{-\infty}^{\infty} F_2(v_x) e^{-ik_{3,z}(v_x)z} e^{iv_x x} \mathrm{d}v_x, \tag{3}$$

where $k_{3,z}(v_x) = \sqrt{(n_3 k_0)^2 - v_x^2}$. Finally, in region IV we have a transmitted component that is a superposition of plane waves propagating away from the sample in the positive z direction:

$$E_{4x} = \int_{-\infty}^{\infty} A_t(v_x) e^{ik_{4,z}(v_x)z} e^{iv_x x} \mathrm{d}v_x, \tag{4}$$

where $k_{4,z}(v_x) = \sqrt{(n_4 k_0)^2 - v_x^2}$. All E_y components are zero due to our choice of geometry and incident polarization. From the free space Maxwell's equations $\nabla \cdot \mathbf{E} = 0$ and $\nabla \times \mathbf{E} = -\mu_0 \partial \mathbf{H} / \partial t$ we obtain the z electric field components, and also the subsequent expressions for the magnetic $\mathbf{H}$-fields.

We now have the electric and magnetic components in all regions of space in terms of six unknow functions $A_r(v_x)$, $G_{1,2}$, $F_{1,2}(v_x)$ and $A_t(v_x)$. We solve for these by applying boundary conditions: the electric fields must be continuous for all x space at the interfaces between adjacent regions,

while the magnetic fields are continuous only over our defined apertures[?,?]. Hence, for the interface between regions 1 and 2 at $z = 0$ we end up with $E_{1x} = E_{2x}$ for the electric and $H_{1y} = H_{2y}$ for the magnetic continuity equations. Substituting eqs. (1) and (2) into the E-field continuity equation for the interface at $z = 0$, and taking its Fourier transform[1], we end up with

$$\delta(\nu_x) + A_r(\nu_x) = (G_1 - G_2)\, Q(\nu_x) \tag{5}$$

where $\delta(\nu_x)$ is the delta function and $Q(\nu_x)$ is the Fourier transform of the rect function, ie.

$$Q(\nu_x) = \frac{1}{2\pi}\int_{-\infty}^{\infty} \mathrm{rect}\left(\frac{x}{w}\right) e^{-i\nu_x x}\mathrm{d}x. \tag{6}$$

The H-field continuity equation at the same interface, $z = 0$, gives

$$k_z(0) - \int_{-\infty}^{\infty} A_r(\nu_x) e^{i\nu_x x} O(\nu_x)\mathrm{d}\nu_x = q_z\,(G_1 + G_2)\,\mathrm{rect}\left(\frac{x}{w}\right) \tag{7}$$

where $O(\nu_x) = \frac{\nu_x^2 + k_{1,z}(\nu_x)^2}{k_{1,z}(\nu_x)}$ in eq. (7). We now substitute $A_r(\nu_x)$ from (5) into (7) and integrate the resulting equation over the values of x for which the magnetic continuity equations hold (the non-conducting regions), obtaining

$$k_z(0)w - \int_{-\infty}^{\infty}\Big((G_1 - G_2)\, Q(\nu_x) - \delta(\nu_x)\Big) I_h(\nu_x) A(\nu_x)\mathrm{d}\nu_x = q_z\,(G_1 + G_2)\, w, \tag{8}$$

where

$$I_h(\nu_x) = \int_{-w/2}^{w/2} e^{i\nu_x x}\mathrm{d}x. \tag{9}$$

Notice that the field amplitudes in the cavities in region 2 do not depend on the directional wavevector ν_x and thus can be taken out of the integral in eq. (8). A similar consideration of the remaining interfaces between the regions is carried out; in the end we obtain six simultaneous equations which are solved for all six amplitude coefficient functions via matrix methods. We can now plot the electric & magnetic fields in any region of space for any choice of parameters $(w, \lambda, n_3, \ldots)$. In doing so, we must numerically evaluate the overlap integrals resulting from these mathematical manipulations. For example, the integral

$$\int_{-\infty}^{\infty} I_h(\nu_x) O(\nu_x) Q(\nu_x)\mathrm{d}\nu_x \tag{10}$$

arising from eq. (8) is numerically evaluated using a Riemann sum over the interval [-125 000, 125 000]m^{-1} with 350 sampling points, each evaluated at the midpoint of the respective subintervals between the sampling points. Note that numerical instabilities were encountered when $\nu_x = n_1 k_0$ since $O(\nu_x)$ diverges to infinity at this point. These instabilities were solved by excluding the values around these poles[2].

Use of model

Our main goal here is to investigate the validity of the permittivity extraction procedure outlined in the main paper. Briefly, this procedure involves solving the Fresenl equations for the transmission through our multilayer system with and without the sample. This is emulated in our model by calculating the far-field transmitted component, i.e. (4) for $\nu_x = 0$, when $n_3 = \sqrt{7.5 + 2i}$ and again when $n_3 = 1$. In both cases we set $n_{1,4} = 1.58$ in order to take into account the effect of the plastic coverslips encapsulating our sample and $n_2 = 3.44$ to model our silicon photomodulator. In our experiments we use a multi-aperture approach, meaning that our final amplitudes result from the addition of fields due to different sized apertures and scatterers. This is also emulated in the model by calculating (4) for a discrete range of values of w and then carrying out a complex summation of these fields, i.e. $\sum_{w_i} E_{4x}(w_i)$. These fields are then processed in a manner similar to the experimentally measured fields, as described in the methods section of the main paper, so as to extract a frequency dependent permittivity of the sample layer. This extracted permittivity is plotted in the main paper as Fig. 2b for three different sample layer thicknesses, and compared to the input value ($n_3 = \sqrt{7.5 + 2i}$) used for the model.

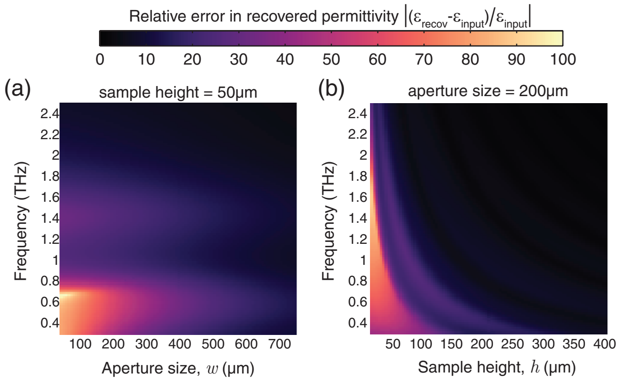

Figure S1. (a) & (b) Colour plots of the percentage error in the recovered permittivity over our frequency regime. In part a) we vary aperture size and keep sample height constant at 50μm, where as in part b) we vary sample height for an aperture size of 200μm.

To further understand the shortcomings of the far-field approximation, in Figs. S1a) & b) we plot the relative error of the recovered permittivity, defined as $|(\varepsilon_{\mathrm{recov}} - \varepsilon_{\mathrm{input}})/\varepsilon_{\mathrm{input}}|$, for our frequency range as we vary aperture size and sample height respectively. These plots show that the permittivity

[1]The Fourier transform is allowed since the E-fields are continuous for all x and the integration equates the fields for all x. However, a Fourier transform of a the H-fields is not possible since they are not continuous for all x space.

[2]To be sure that excluding the poles did not affect the output value, we used the various intergration algorithms built in to Wolfram Mathematica 9 to obtain consistent values between all these algorithms. The Riemann sum was chosen for the final evaluation due to its speed.

extraction procedure becomes more applicable as aperture size and sample thickness are increased. In Fig. S1a) we see two frequency independent maxima centered around 0.6 and 1.4THz, the strengths of which decrease with aperture size. In part b) we see the frequency of these same maxima changing with sample height. These maximal errors arise at the Fabry-Perot resonances of the system. The far-field approximation used in permittivity extraction miscalculates these resonant frequencies due to the presence of evanescent fields, which change the impedance of each interface.

S2 Raw time data and Absorption maps

As outlined in the main paper, by varying the arrival time of the optical detection pulse we measure the temporal evolution of our THz pulse. We, however, take images at each temporal point resulting in a hypertemporal stack of images. These stacks are shown in supplementary videos S1 and S2 where we present the raw data used to extract the permittivity of the cartilage sample for horizontal and vertical polarization, respectively. A pair of still images from the two videos are shown in Fig. S2. These stacks are Fourier transformed along their temporal direction to obtain a hyperspectral stack and further analyzed as outlined in the main paper to obtain the permittivity of the cartilage sample.

From these hypertemporal stacks one can obtain the frequency response of each pixel and then obtain the permittivity at each point in space, as outlined in the methods sections of main manuscript. In figs. S3(a) and (b) we show an example of these absorption maps when the sample is aligned horizontally and vertically respectively across the field of view. The main manuscript shows an optical image of a horizontally aligned sample in fig. 3(a). Note that our electric field polarization is horizontal in both absorption maps. In these images we can see a gradient in the absorption. This gradient was found to arrise from misalignment in the sample and reference measurements. In other words, between the sample and reference measurements there might be a 1° (in both x and y) different angle of incidence on to the sample. In order to remove this gradient effect, we plot the permittivity as averaged horizontally and vertically across our absorption maps. Since the sample has an inhomogeneity only across 1-dimension (see fig. 3 in the main manuscript), we can still look at the near-field permittivity across the inhomogeneity. This is what we plot for all frequencies in figs. S3(c)-(f), where parts (c) and (d) are averaged parallel to the sample (shown by the coloured rectangles in parts (a) and (b) respectively) and parts (e) and (f) are averaged perpendicularly to the sample. We can see that the parallel averaging has a clear dependance on position where as the perpendicular averaging does not have anything obvious. This is what we expect as the parallel averaging is taken from different inhomogenous areas of the sample.

S3 Second sample measurements

For comparison, we present the permittivities measured for second sample in fig. S4 (windowed with a Chebyshev window function), where the colour coding is the same as the main manuscript. We observe similar features as the previous sample; namely, when the THz polarization is horizontal (vertical) the real (imaginary) part of the THz permittivity increases (decreases) going from the superficial to the deep zone (from top to bottom in fig. 3a). Further, when the polarization is rotated by 90° this behaviour is reversed. It is important to note that slight variations in the sample thickness and hydration causes variation in the extracted magnitudes of the real and imaginary parts of the permittivity, a well known problem in phase resolved measurements. Nevertheless, in all data sets we observe the same resonant feature around $\sim 1.6THz$ in regions where the polarization is aligned to the fibril axis.

References

1. J. W. Goodman *Introduction to Fourier Optics* Roberts & Company Publishers, 3rd ed. (2005) pp. 55-73

2. Marek W. Kowarz *Homogeneous and evanescent contributions in scalar near-field diffraction* Applied Optics, Vol. 34 (1995)

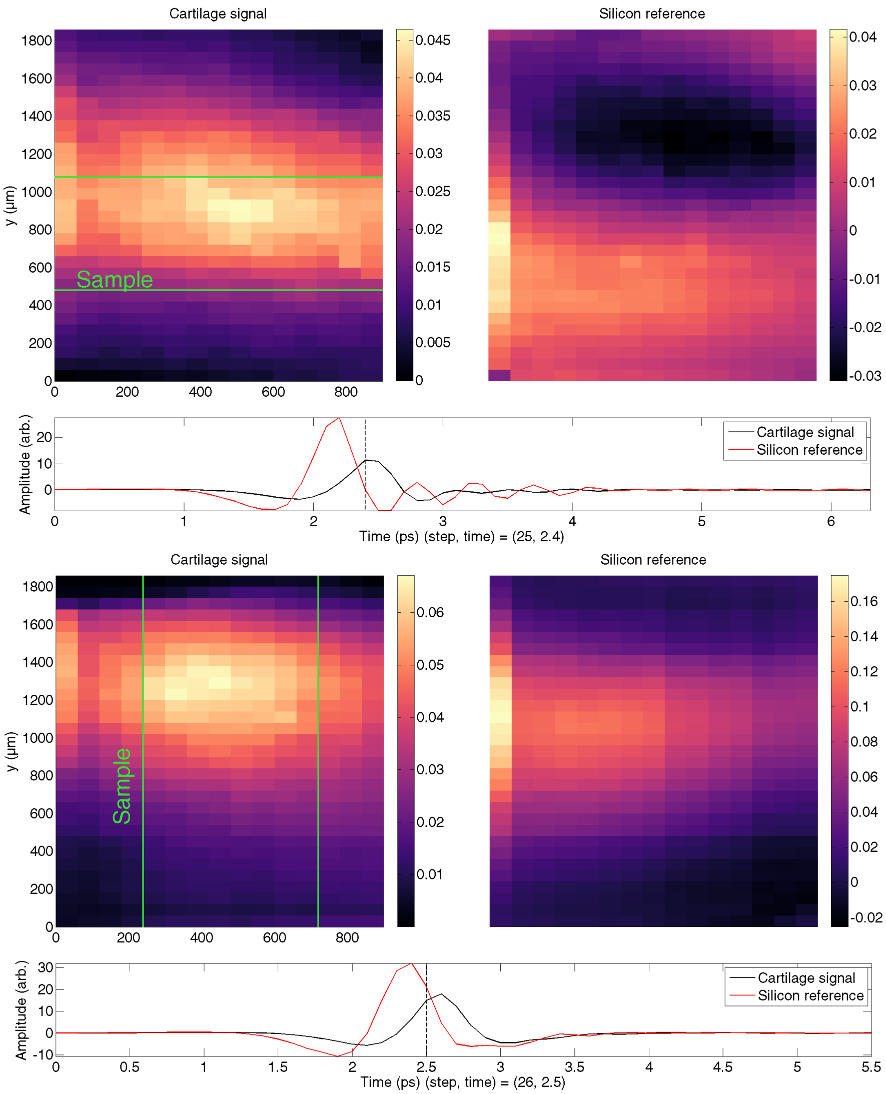

Figure S2. Still images from supplementary videos S1 and S2 on the top and bottom, respectively. In both cases, a temporal trace of our averaged signals is shown below the two colourmaps. A vertical dashed line shows the time at which the above colourmaps have been taken at ($\sim 2.5ps$). The location of the sample is indicated by the green lines on left colourmap.

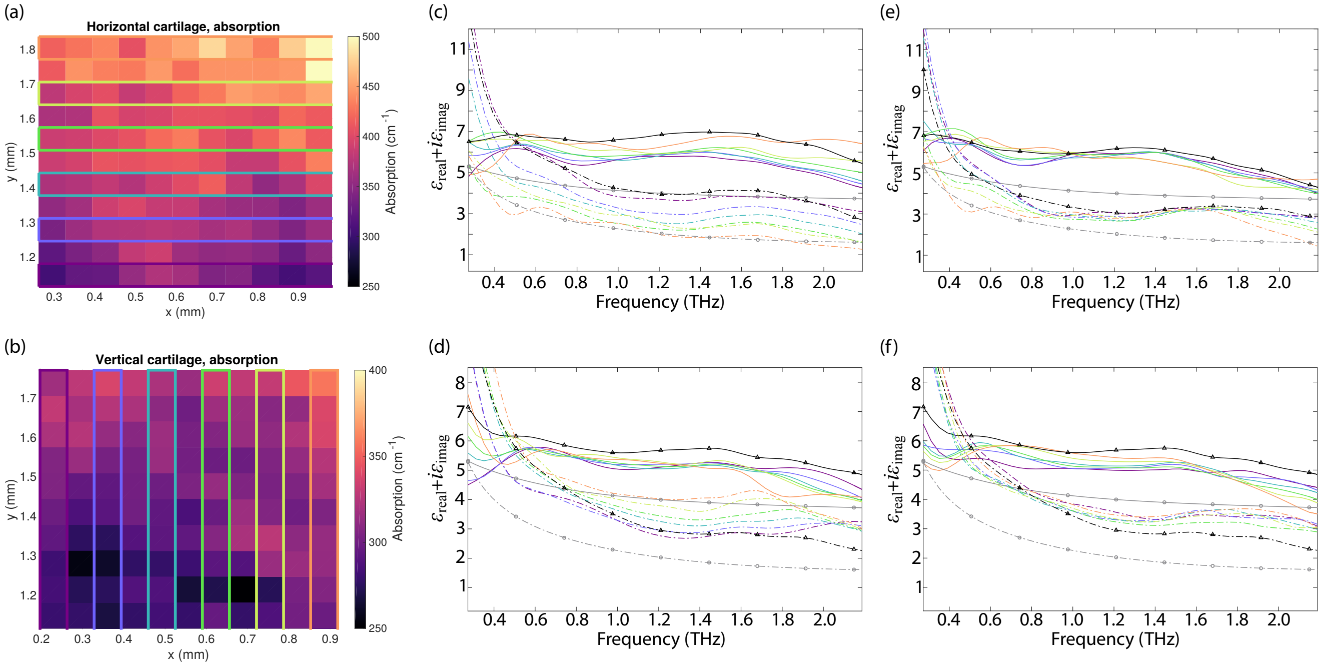

Figure S3. (**a**) & (**b**) Absorption maps at 1THz when the cartilage sample is aligned horizontally & vertically respectively across the field (see fig. 3(a) for an optical image of a horizontally aligned sample). (**c**) & (**d**) The permittivity when it is averaged parallel to the sample alignment (shown the by coloured rectangles in parts (a) and (b)). These are the results of figs. 3(b-e) shown in the manuscript. (**e**) & (**f**) The permittivity when it is averaged perpendicularly to the sample alignement. In parts (c) to (f) the real (imaginary) permittivity is shown in the solid (dash-dotted) line. The black and grey lines are respectively the far-field and water response.

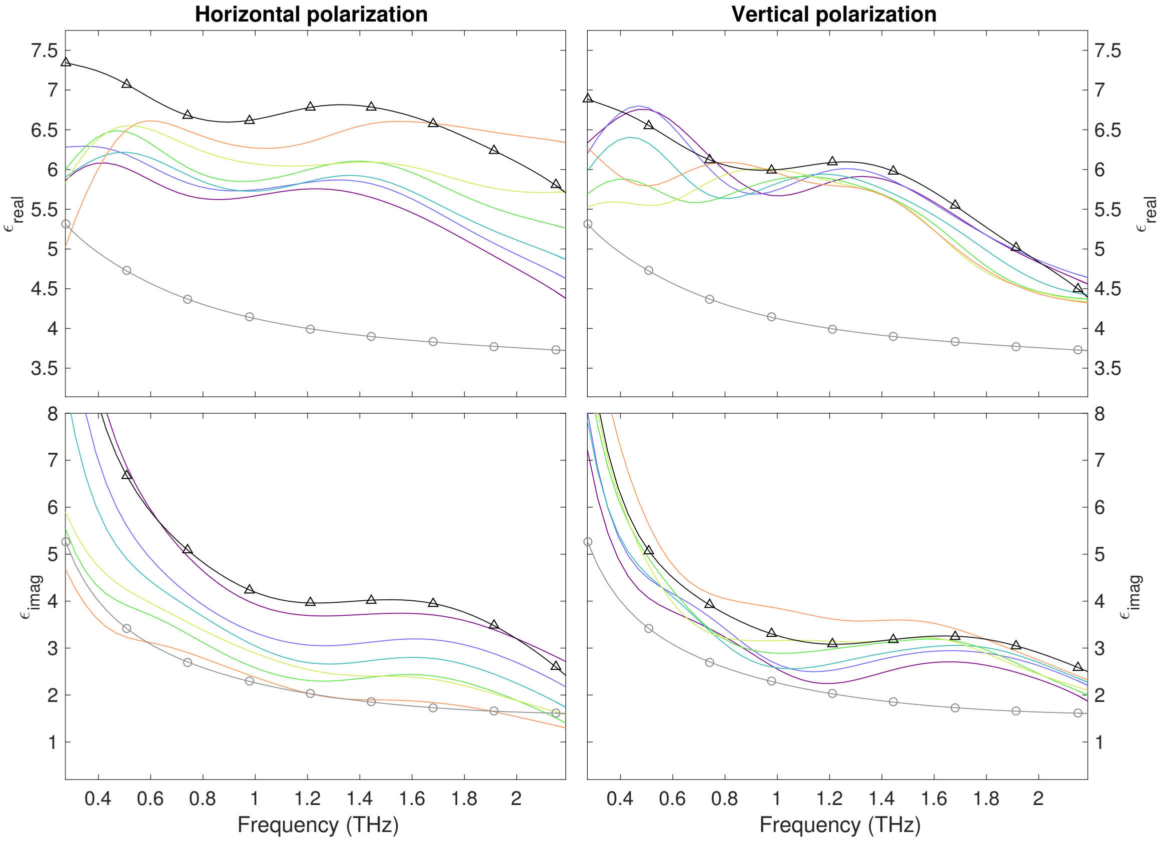

Figure S4. Permitivity measurement of supplementary sample. Colour coding is the same as fig. 3 in the main manuscript, ie. deep purple is the top part of the sample with fibrils aligned horizontally and going to green to orange the fibrils rotate their orientation by 90° to end up orientated vertically.
